# Local, semi-automatic, three-dimensional liver reconstruction or external provider? An analysis of performance and time expense

**DOI:** 10.1007/s00423-020-01862-7

**Published:** 2020-03-25

**Authors:** Markus Paschold, Florentine Huettl, Werner Kneist, Christian Boedecker, Alicia Poplawski, Tobias Huber, Hauke Lang

**Affiliations:** 1 Department of General, Visceral and Transplant Surgery, University Medical Center of the Johannes Gutenberg-University Mainz, Langenbeckstraße 1, 55131 Mainz, Germany; 2 Institute of Medical Biostatistics, Epidemiology and Informatics (IMBEI), University Medical Center of the Johannes Gutenberg-University Mainz, Mainz, Germany

**Keywords:** Liver surgery, 3D segmentation, Surgical planning, Virtual surgery, Surgical education

## Abstract

**Purpose:**

In hepatobiliary surgery, preoperative three-dimensional reconstruction based on CT or MRI can be provided externally or by local, semi-automatic software. We analyzed the time expense and quality of external versus local three-dimensional reconstructions.

**Methods:**

Three first-year residents reconstructed data from 20 patients with liver pathologies using a local, semi-automatic, server-based program. Initially, five randomly selected patient datasets were segmented, with the visualization of an established external company available for comparison at all times (learning phase). The other fifteen cases were compared with the external datasets after completing local reconstruction (control phase). Total time expense/case and for specific manual and semi-automated reconstruction steps were recorded. Segmentation quality was analyzed by testing the equivalence for liver and tumor volumes, portal vein sectors, and hepatic vein territories.

**Results:**

The median total reconstruction time was reduced from 2.5 h (learning phase) to 1.5 h (control phase) (− 42%; *p* < 0.001). Comparing the total and detailed liver volumes (sectors and territories) as well as the tumor volumes in the control phase equivalence was proven. In addition, a highly significant correlation between the external and local analysis was obtained over all analyzed segments with a very high ICC (median [IQR]: 0.98 [0.97; 0.99]; *p* < 0.01).

**Conclusion:**

Local, semi-automatic reconstruction performed by inexperienced residents was feasible with an expert level time expense and the quality of the three-dimensional images was comparable with those from an external provider.

## Introduction

During hepatobiliary surgery, knowledge of the tumor’s localization, relationship to vascular structures, and the functional liver remnant after resection are essential [1, 2]. For complex resections such as extended hepatectomy or major vessel reconstruction, where the limits of resectability are approached, the visualization of critical structures that must be preserved can be demanding, especially since only 29% of patients have standard liver anatomy [3]. In laparoscopic liver resection, knowledge of individual variability is of even higher importance. The imagination of the intraoperative situation from preoperative two-dimensional imaging can be quite difficult in these situations.

Three-dimensional liver reconstruction is a useful tool for visualizing critical structures for surgical planning and intraoperative orientation [3, 4]. Furthermore, it is the basis for three-dimensional printing and computer-assisted navigation [5, 6].

Usually, established external providers create such complete segmentations [7]. However, there is an increasing availability of local server-based segmentation programs. The aim of this study was to evaluate the time expense and the quality of local reconstruction, performed by inexperienced residents, with the external reconstructions.

## Methods

### Study design

We included 20 consecutive patients with an external preoperative three-dimensional reconstruction available independently from the current study. The same established provider (MeVis Medical Solutions AG, Bremen, Germany) performed all external reconstructions. The three-dimensional images were based on computed tomography (CT) scans. All patients had liver tumors that were resected using an open or laparoscopic approach.

For local reconstruction, a semi-automatic, server-based program (Synapse 3D, FUJIFILM Europe GmbH, Düsseldorf) was used. Three first-year residents of the surgical department, who had no expert knowledge in the fields of liver surgery, radiology, or informatics prior to the study, performed these reconstructions. In preparation, the residents completed a half-day training session at the radiology department’s CT unit under the direction of a radiological consultant to train export functions and the general handling of two-dimensional datasets. The distributor provided a one-time, 2-h training session on the planning software tool.

Patient identifiers (1–20) were assigned randomly. The first five patients’ datasets were reconstructed, with the external visualization available for comparison at all times (learning phase). The other cases (*n* = 15) were compared with the corresponding external reconstruction after the three-dimensional image was completed (control phase).

### External reconstruction

MeVis Medical Solutions is an internationally recognized research and development center for medical image computing closely collaborating with *Frauenhofer MEVIS*. Several studies have proven the accuracy of the visualization by this provider [8–11].

The CT datasets for each patient were exported as DICOM files, anonymized, and sent to the company. Scans had to be delivered with a quality of 0.8- to 1.5-mm slice increment and with contrasted portal and hepatic veins that were at least 30 Hounsfield units above liver parenchyma. Regular priority visualization takes 3 days, and data were presented as a three-dimensional PDF file that displayed calculated volumes for the liver parenchyma, tumor, portal vein (PV), and hepatic vein (HV) systems.

### Local reconstruction

The authors tested different semi-automatic and completely manual software tools for three-dimensional liver reconstruction prior to this study. The mentioned program was acquired for the department independently from the current study.

The patients’ exported DICOM files were uploaded to a local server. The program automatically extracts the raw circumference of the liver parenchyma and the vessels (PV and HV), which needed to be checked and corrected manually. The circumference of the inferior vena cava (IVC) must be traced manually in several slices, and the program interpolates in between. For tumor reconstruction, the largest diameter was marked by hand. Before the three-dimensional model was created, the direction of blood flow in the PV and HV was defined by setting a root point. The feeding vessels were selected manually for the division of the liver parenchyma into different segments and sectors. The program then calculated the volumes automatically.

### Segmentation time analysis

The time of reconstruction was recorded for each individual case (total reconstruction time). Furthermore, the duration of each step in visualizing the liver was measured. This included manual editing of the automatically extracted liver parenchyma, IVC, PV, HV, and tumor (step 1, semi-automatic). In addition, the time needed to complete individual segment and territory analysis with optimization of blood vessel visualization was recorded (step 2, fully manual).

### Liver volume analysis

We compared the volumes of the liver, tumor, segments (PV), sectors (PV), and territories (HV) of the local and external three-dimensional analyses to objectify one aspect of the quality of the three-dimensional reconstruction.

### Statistical analysis

For case number planning, the SAS software (SAS Institute GmbH, Heidelberg, Germany) was used. A minimum case number of 13 was calculated for the control phase applying a lower and upper equivalence bound of 7.5% and a power of 80% based on available literature [12, 13]. Further statistical analyses were performed with IBM SPSS Statistics 23 (IBM, Armonk, NY, USA). The standardized difference was calculated using the following equation:

$$ \left({\mathrm{volume}}_{\mathrm{external}}-{\mathrm{volume}}_{\mathrm{local}}\right)/{\mathrm{volume}}_{\mathrm{external}}=\Delta {\mathrm{volume}}_{\mathrm{standardized}} $$

For equivalence testing, the 95% confidence level of the one sample *t* test was calculated. A deviation of more than 0.075 was set as equivalence limit. The intraclass correlation coefficient (ICC) was used to correlate volumes. Nonparametric Mann-Whitney *U* test was performed to analyze time differences. *P* values were adjusted for multiple testing using the Benjamini and Hochberg (FDR) method. *P* values < 0.05 were considered statistically significant. Data are presented as the median and interquartile range (IQR).

## Results

Patient’s characteristics are displayed in Table 1. The CT data used were from patients with various resectable pathologies and represent very different types of hepatectomy.

**Table 1:** Characteristics of selected patients (*n* = 20)

Characteristics	Value
Age (years, mean (range))	62 (22–79)
Gender	
Male	13 (65%)
Female	7 (35%)
Underlying disease	
Colorectal liver metastases	10 (50%)
Hepatocellular carcinoma	7 (35%)
Focal nodular hyperplasia	1 (5%)
Breast cancer metastases	1 (5%)
Echinococcus multilocularis	1 (5%)
Operation type	
Laparoscopic	8 (40%)
Open	12 (60%)
Type of resection	
Minor resection	
Non-anatomical resection	4 (20%)
Segmentectomy	10 (50%)
Bisegmentectomy	2 (10%)
Major resection	
Left hemihepatectomy	1 (5%)
Extended left hemihepatectomy	3 (15%)

### Segmentation time analysis

**Fig. 1:**
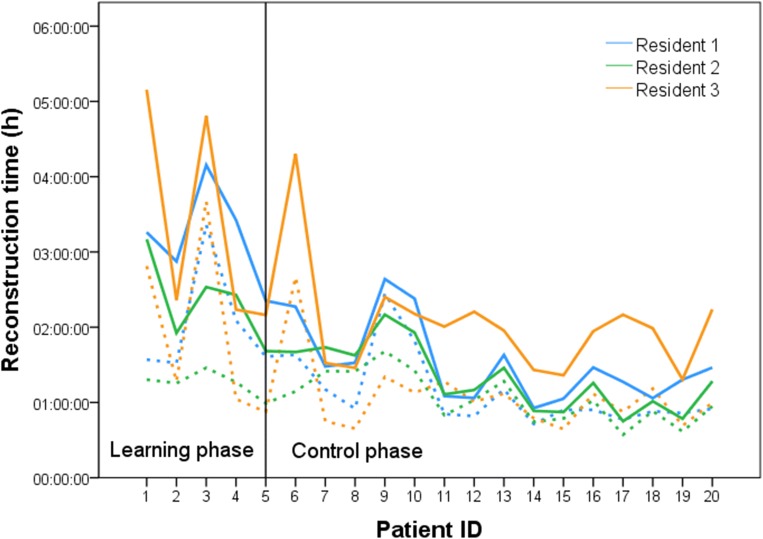
Reconstruction time of the CT scans from all patients. Solid lines represent total reconstruction time. Dashed lines represent the time for reconstruction step 1

Figure 1 displays the time for step 1 and the total reconstruction time required for editing the data of all patients for each resident. After an initial median total reconstruction time of 2.5 h (learning phase), the duration was reduced to a median level of 1.5 h (control phase) (− 42%; *P* < 0.001). The median time and IQR for the different reconstruction steps in total and for each resident are shown in Table 2. Between learning and control phase timesavings up to 81% (resident 2) could be achieved for reconstruction step 2.

**Table 2:** Reconstruction time

	Learning phase	Control phase	Difference	
	Time (min)	Time (min)	Time (min)	
	Median (IQR)	Median (IQR)	Median (%)	*P*^1^
Resident 1				
Reconstruction step 1	97 (93;163)	54 (51;70)	43 (44)	0.019*
Reconstruction step 2	80 (46;91)	27 (13;34)	53 (66)	0.001*
Total reconstruction time	196 (157;227)	88 (64;158)	108 (55)	0.002*
Resident 2				
Reconstruction step 1	75 (62;77)	62 (47;85)	13 (17)	0.407
Reconstruction step 2	70 (41;100)	13 (9;19)	57 (81)	0.001*
Total reconstruction time	146 (108;171)	76 (53;100)	70 (48)	0.003*
Resident 3				
Reconstruction step 1	68 (58;123)	61 (45;68)	7 (10)	0.182
Reconstruction step 2	77 (68;181)	50 (44;74)	27 (35)	0.035*
Total reconstruction time	141 (132;299)	119 (88;132)	22 (15)	0.023*
Total				
Reconstruction step 1	77 (64;97)	57 (48;71)	20 (26)	0.002*
Reconstruction step 2	77 (48;102)	29 (13;45)	48 (62)	< 0.001*
Total reconstruction time	152 (134;206)	88 (68;120)	64 (42)	< 0.001*

Learning phase: external reconstruction available for comparison; control phase: external reconstruction not available for comparison. *IQR*, interquartile range

^1^Adjusted for multiple testing

*Significant

### Liver volume analysis

Comparing the total and detailed liver volumes during the control phase, a high intraclass correlation coefficient was achieved over all analyzed volumes (median ICC [IQR]: 0.98 [0.97; 0.99]; *p* < 0.01) (Table 3). Equivalence was proven for all three residents in the control phase for tumor volume, total liver volume, left and right portal vein, and the lateral and medial sectors. Liver segment and right sector analysis revealed a higher variability and did thus reach different levels of equivalence for the three residents (Table 3).

**Table 3:** Quality assessment of the control phase (*n* = 15): comparison of liver volume results

	Correlation		One sample *t* test		
	ICC	*P*^1^	Resident 1	Resident 2	Resident 3
			95% CI	95% CI	95% CI
Liver	0.998	< 0.01*	*[− 0.022;− 0.008]*	*[− 0.030;0.012]*	*[− 0.017;− 0.004]*
Tumor	0.998	< 0.01*	*[− 0.019;0.060]*	*[− 0.062;0.013]*	*[− 0.061;0.026]*
Left PV	0.994	< 0.01*	*[− 0.039;− 0.003]*	*[− 0.054;0.058]*	*[− 0.050;0.028]*
Right PV	0.990	< 0.01*	*[− 0.011;0.036]*	*[− 0.017;0.062]*	*[− 0.025;0.013]*
Lateral PV	0.997	< 0.01*	*[− 0.046;0.014]*	*[− 0.075;0.043]*	*[− 0.040;0.030]*
Medial PV	0.992	< 0.01*	*[− 0.058;0.039]*	*[− 0.069;0.060]*	*[− 0.096;0.025]*
Anterior PV	0.972	< 0.01*	*[− 0.001;0.069]*	[− 0.068;0.137]	*[− 0.047;0.030]*
Posterior PV	0.981	< 0.01*	[− 0.084;0.042]	[− 0.106; 0.053]	*[− 0.045;0.033]*
Segment 1	0.976	< 0.01*	[− 0.134;0.146]	[− 0.303;*−* 0.032]	[− 0.194;0.127]
Segment 2	0.997	< 0.01*	[− 0.146;*−* 0.013]	[− 0.255;*−* 0.021]	*[− 0.053;0.010]*
Segment 3	0.988	< 0.01*	[− 0.143;0.097]	[− 0.040;0.141]	*[− 0.046;0.060]*
Segment 4a	0.933	< 0.01*	[− 0.215;0.029]	[− 0.034;0.338]	[− 0.108;0.043]
Segment 4b	0.922	< 0.01*	[− 0.335;0.153]	[− 0.829;0.002]	[− 0.076;0.081]
Segment 5	0.937	< 0.01*	[− 0.039;0.131]	[− 0.424;0.400]	[− 0.197;0.116]
Segment 6	0.958	< 0.01*	[− 0.174;0.048]	[− 0.272;0.129]	*[− 0.061;0.074]*
Segment 7	0.976	< 0.01*	[− 0.100;0.049]	[− 0.411;0.169]	*[− 0.063;0.064]*
Segment 8	0.976	< 0.01*	*[− 0.031;0.048]*	[− 0.128;0.057]	[− 0.109;0.002]
Left HV	0.992	< 0.01*	*[0.065;0.014]*	*[− 0.075;0.063]*	*[− 0.048;0.012]*
Middle HV	0.976	< 0.01*	*[− 0.012;0.041]*	*[− 0.014;0.025]*	*[− 0.035;0.010]*
Right HV	0.995	< 0.01*	*[− 0.045;0.011]*	*[− 0.040;0.058]*	*[− 0.032;0.020]*

*CI*, confidence interval. Values in italics are proven equivalence

^1^Adjusted for multiple testing

*Significant

### Liver segmentation

Due to previous resections (right hemihepatectomy) in one case segments 5 to 8 could not be reconstructed. In three cases, segment vessels were displaced by the tumor (twice segment 1, once segment 4a, and three times segment 4b). Poor vascular contrast was responsible for not identifying all segments in three cases: twice segment 1, once segment 4b, and once segment 5. In addition, resident 1 could not identify the feeding vessel once for segment 2 and for segment 5. The later was also not identified by resident 2. In the two additional cases with the local software, the tumor was closely adjacent to the missing segment.

## Discussion

The benefit of three-dimensional visualizations, especially for preoperative planning of complex and extended liver resections, has been analyzed previously [4, 14]. This is the first study describing the feasibility and quality of a semi-automatic, local, server-based program for three-dimensional liver reconstruction under the premise that first-year residents performed all reconstructions.

**Fig. 2:**
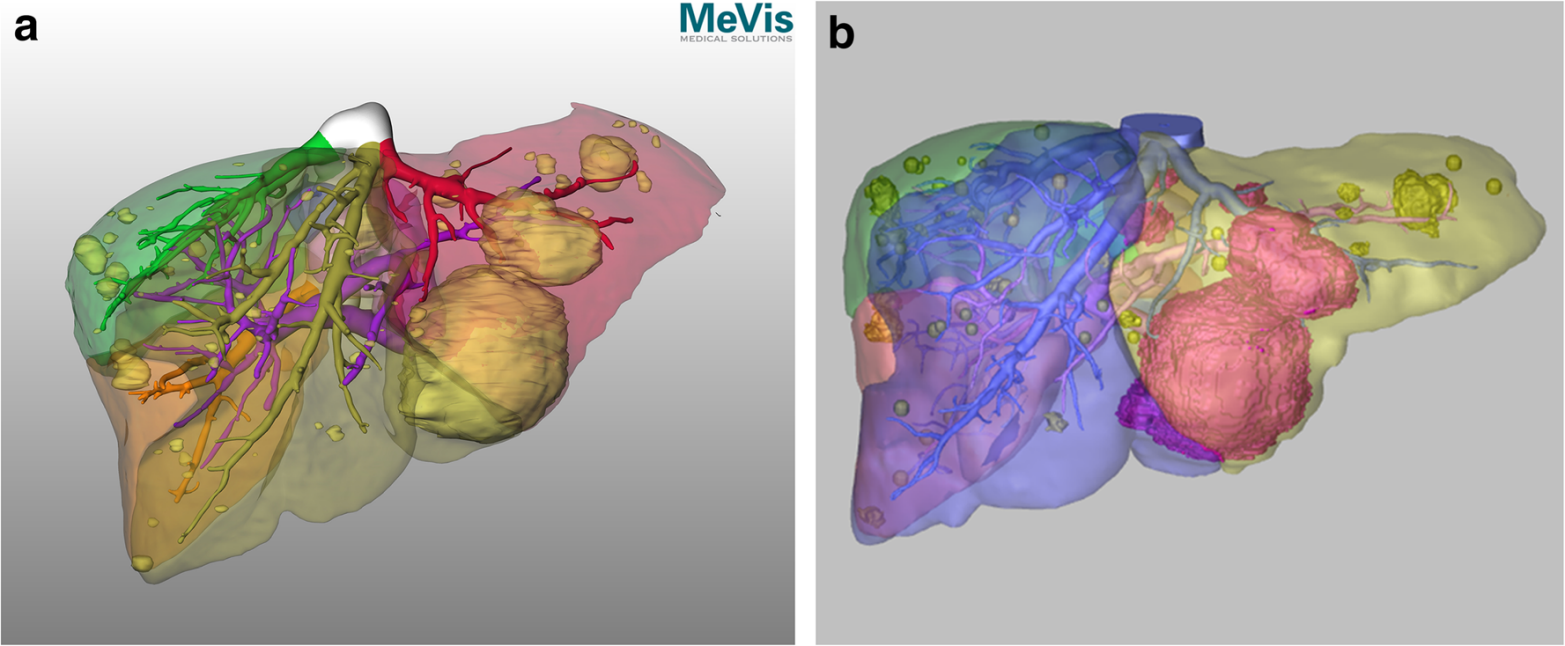
Liver reconstruction (Pat. ID 10). External reconstruction (**a**): Three-dimensional visualization of a patient’s liver with five breast cancer metastases and multiple intrahepatic cystic lesions (yellow). Portal vein (pink) and hepatic vein (color coded) are visualized. In this figure, hepatic vein territories including an inferior right hepatic vein are color coded. Image courtesy of © 2019 MeVis Medical Solutions AG, Bremen, Germany. Local reconstruction (**b**): Three-dimensional visualization of a patient’s liver with five breast cancer metastases (purple) and multiple intrahepatic cystic lesions (yellow). Portal vein (pink) and hepatic vein (blue) are visualized. In this figure, hepatic vein territories including an inferior right hepatic vein are color coded.

One aspect of this study was to investigate the residents’ time expense in comparison with highly specialized experts for generating the reconstruction. The median time to complete the reconstruction of the patients’ CT scan data was 2,5 h in the learning phase, but was reduced to 1.5 h during the control phase. This is comparable with the work of Endo et al. [1], with a similar total reconstruction time of about 2 h. Two hours is also considered an expert level by Lang et al. [4]. Sboarina et al. stated that with improved skill, the time for liver and vessel segmentation can be reduced by 30–40% [15]. This can be confirmed in our study, when comparing the learning phase to the control phase. The editing time needed for step 1, step 2, and total reconstruction was reduced by 26%, 62%, and 42% respectively. The greatest time saving could be shown for step 2 (fully manual), representing a steep learning curve for all residents. Step 1 (semi-automatic) shows the least time reduction and variations in total reconstruction time are highly dependent on this part of the process. In three patients (3, 9, and 10), the time required for editing during step 1 was longer for all residents due to a necessary alteration in the extracted liver anatomy. In patient 3, the liver parenchyma was not automatically extracted, resulting in the manual editing of the liver data and a longer total reconstruction time, respectively. Furthermore, intraparenchymal HV was traced as the PV, making manual alterations necessary. The reason may be that the patient had steatosis hepatis, which is known to attenuate Hounsfield units [16, 17]. In patient 9, a large tumor influenced the contrasting of the PV, HV, and IVC. In addition, an adjacent lymph node was registered as liver parenchyma. Patient 10 had multiple intrahepatic cystic lesions next to breast cancer metastases, which required extensive manual alteration of the liver data (Fig. 2). To sum up, we could demonstrate that residents can perform three-dimensional liver reconstruction within a comparable time expense to experts after a reasonable time expense for training.

Furthermore, we focused on the quality of the liver reconstruction. This is in contrast to the previously cited studies, which measured the general time expense without analyzing the quality of the reconstruction. A very high ICC above 0.9 for all analyzed volumes and a proven equivalence for all sectors and territories demonstrated a good quality of liver segmentation images in the current study. The variance in segmental volumes can be explained by the naturally smooth transitions between segments in general and very thin vessels at the end of the branches [18]. In areas with very small vessels that are difficult to reconstruct especially segment 1 and segment 4a/b, a higher variation was observed. The selection of the feeding vessel and the associated liver volume can also be subjected to human errors and subjective interpretation [18]. Especially for the right hepatic lobe, the assignment of smaller vessels at the borders of the segments can be demanding. On the other hand, the calculation of the parenchyma volume to the feeding vessel is depending on the underlying software and can therefore variate between the external and local reconstructions. The clinical relevance of this variation is questionable.

A limitation of this study is the fact that only surgical residents from one center used the program. Further studies should investigate if quality and time expenses of three-dimensional reconstruction vary by participants with different medical specialties and from different centers. The comparison between several local software providers may also be of interest.

From an economical point of view, annual service costs aside from the initial acquisition price must be considered when using a local segmentation program. In our setting, 45–50 cases are needed to amortize the initial costs. Annual costs will be equalized at about 10 reconstructions per year. In addition, the calculations must include around 2–3 days of a residents working time for the training phase and around 2 h per case. In our opinion, this results in approximately one additional case for the initial costs amortization and one additional case per year to create financial compensation. However, the benefits of a profound knowledge of patients’ individual liver anatomy by performing the reconstruction locally are difficult to put into numbers. The general benefit of a local segmentation program is that the reconstruction is available faster than the usual 3-day time expense for external visualization that is invoiced per case. Thus, a center must evaluate whether the investment of a local reconstruction program is suitable and economic according to its caseload for complex liver surgery.

## Conclusion

In conclusion, this study reveals that local, semi-automatic reconstruction is feasible and comparable with an external provider regarding the total and detailed volumes when performed by an inexperienced resident after a reasonable amount of training. Variation in the time expense for reconstructions is currently due to necessary manual corrections of the automated part of the analysis. The software may be optimized to offer different choices in uncertain cases that may improve the automated reconstruction process in the future (deep learning).
